# Micro-ultracapacitors with highly doped silicon nanowires electrodes

**DOI:** 10.1186/1556-276X-8-38

**Published:** 2013-01-21

**Authors:** Fleur Thissandier, Nicolas Pauc, Thierry Brousse, Pascal Gentile, Saïd Sadki

**Affiliations:** 1 Laboratory for Molecular Electronics Organic & Hybrid (LEMOH)-SPrAM UMR 5819 (CEA,CNRS, UJF), Grenoble, 38054, France; 2 SiNaPS Laboratory SP2M, UMR-E CEA/UJF-Grenoble 1, Grenoble, 38054, France; 3 Commisioner for Atomic Energy and Alternative Energies (CEA) Grenoble/INAC 17 Rue des Martyrs, Grenoble Cedex 9, 38054, France; 4 Institut des Matériaux Jean Rouxel (IMN), Université de Nantes, CNRS, 2 Rue de la Houssinière, BP32229, Nantes, Cedex 3, 44322, France

**Keywords:** Ultracapacitor, Electrochemical capacitor, Silicon nanowires electrodes, Chemical vapor deposition, Cyclic voltammetry, Galvanostatic charge/discharge, Microdevice

## Abstract

Highly n-doped silicon nanowires (SiNWs) with several lengths have been deposited via chemical vapor deposition on silicon substrate. These nanostructured silicon substrates have been used as electrodes to build symmetrical micro-ultracapacitors. These devices show a quasi-ideal capacitive behavior in organic electrolyte (1 M NEt_4_BF_4_ in propylene carbonate). Their capacitance increases with the length of SiNWs on the electrode and has been improved up to 10 μFcm^−2^ by using 20 μm SiNWs, i.e., ≈10-fold bulk silicon capacitance. This device exhibits promising galvanostatic charge/discharge cycling stability with a maximum power density of 1.4 mW cm^−2^.

## Background

Nowadays, electronic devices invade strongly our daily life. In the race to efficiency, they have to be faster and faster, smaller and smaller, and with better and better performance [1-4]. One way to reach this goal is to integrate supercapacitors in their microelectronic circuit. Supercapacitors are commonly used to complete batteries whenever pulse power, long term cycling, and high charge/discharge are required [5-9]. Many studies are currently dedicated to the design of micro-ultracapacitors with different types of carbons [5-7] or pseudo-capacitive materials (RuO_2_, MnO_2_ …) [8,9]. However, their integration in microelectronic circuit is still a challenge. Elaborate silicon based micro-ultracapacitors should facilitate it. Moreover, such devices could directly be manufactured on chips. Recently, porous silicon nanowires (SiNWs) [10], porous silicon coated with gold [11,12], SiNWs coated with NiO [13,14], or SiC [15] have been studied as potential materials for supercapacitor electrodes. Si/SiC core-shell nanowires-based electrodes show the most promising performances and cycling stability, but no studies have been performed in the two electrode devices. More recently, we proved that chemical vapor deposition (CVD)-grown, SiNWs-based electrodes show a promising cycling stability in an organic electrolyte and a quasi-ideal pure capacitive behavior, i.e., the energy that is stored thanks to electrolyte ions accumulation at the polarized electrode/electrolyte interface [16]. As pure capacitive supercapacitor capacitance is proportional to the developed surface area on the electrode, increasing the SiNWs length should improve the device capacitance. SiNWs length and doping level can easily be tuned by CVD, thanks to the vapor–liquid-solid (VLS) mechanism [17,18], using a metal catalyst as seed to the SiNWs growth [19-21]. The SiNWs diameter and density can also be monitored.

This work underlines the importance of HCl use during the SiNWs growth by CVD to obtain very long nanowires and investigates the influence of SiNWs length on SiNWs/SiNWs micro-ultracapacitors devices capacitance.

## Methods

### Doped silicon nanowires growth

Several SiNWs electrodes with each different SiNWs length have been made. The SiNWs were grown in a CVD reactor by VLS method via gold catalysis on highly doped n-Si (111) substrate (doping level (i.e., the number of doping atoms per cubic centimeter of materials, *N*_d_ = 5.10^18^ cm^−3^). Gold colloids with size of 50 nm are used as catalysts, H_2_ as carrier gas, silane (SiH_4_) as silicon precursor, phosphine (PH_3_) as n-doping gas, and HCl as additive gas. As shown in our previous work [19-21], the use of HCl in our process enables us to reduce the gold surface migration. Thus, the nanowires (NWs) morphology is improved and their length is not limited.

Prior to the growth, the substrates surface has been prepared by successive dipping in (a) acetone, isopropanol and caro (H_2_SO_4_/H_2_O_2_, 3:1) to remove organic impurities followed by (b) 10% HF and NH_4_F solution to remove the native oxide layer. Then, 50-nm gold colloids are deposited on the surface with 10% HF from an aqueous gold colloid solution (British Bio Cell International Ltd., Llanishen, Cardiff, UK).

The growth has been performed at 600°C, under 3 Torr total pressure, with 40 sccm (standard cubic centimeters) of SiH_4_, 100 sccm of PH_3_ gas (0.2% PH_3_ in H_2_), 100 sccm of HCl gas and 700 sccm of H_2_ as supporting gas [19]. Our VLS-CVD method enables an easier control of SiNWs parameters (length, density, diameter, doping type, and doping level) and growth on low cost substrates. The doping level of the SiNWs is managed by the pressure ratio: dopant gas/SiH_4_. In our setup the ratio can vary from 10^−6^ to 10^−2^ to obtain doping level from Nd ≈10^16^ to ≈10^20^ cm^−3^[20]. It was checked by resistivity measurements in four probes configuration [21,22]. The SiNWs length is monitored by the gas injection time. The growth rate is about 500 nm/min under these conditions.

SiNWs morphologies are checked by scanning electron microscopy (SEM) before and after electrochemical cycling. SiNWs density is estimated by counting the number of gold colloids per square centimeters on several SEM images.

### SiNWs electrochemical characterization

All experiments were performed in a glove box at room temperature. The electrolyte was 1 M NEt_4_BF_4_ (Fluka Chemika, Buchs, Switzerland) in propylene carbonate (Sigma Aldrich, St. Louis, MO, USA). Nanostructured silicon (n-SiNWs) and bulk silicon substrates (n-Si) were always directly used as electrodes.

**Figure 1:**
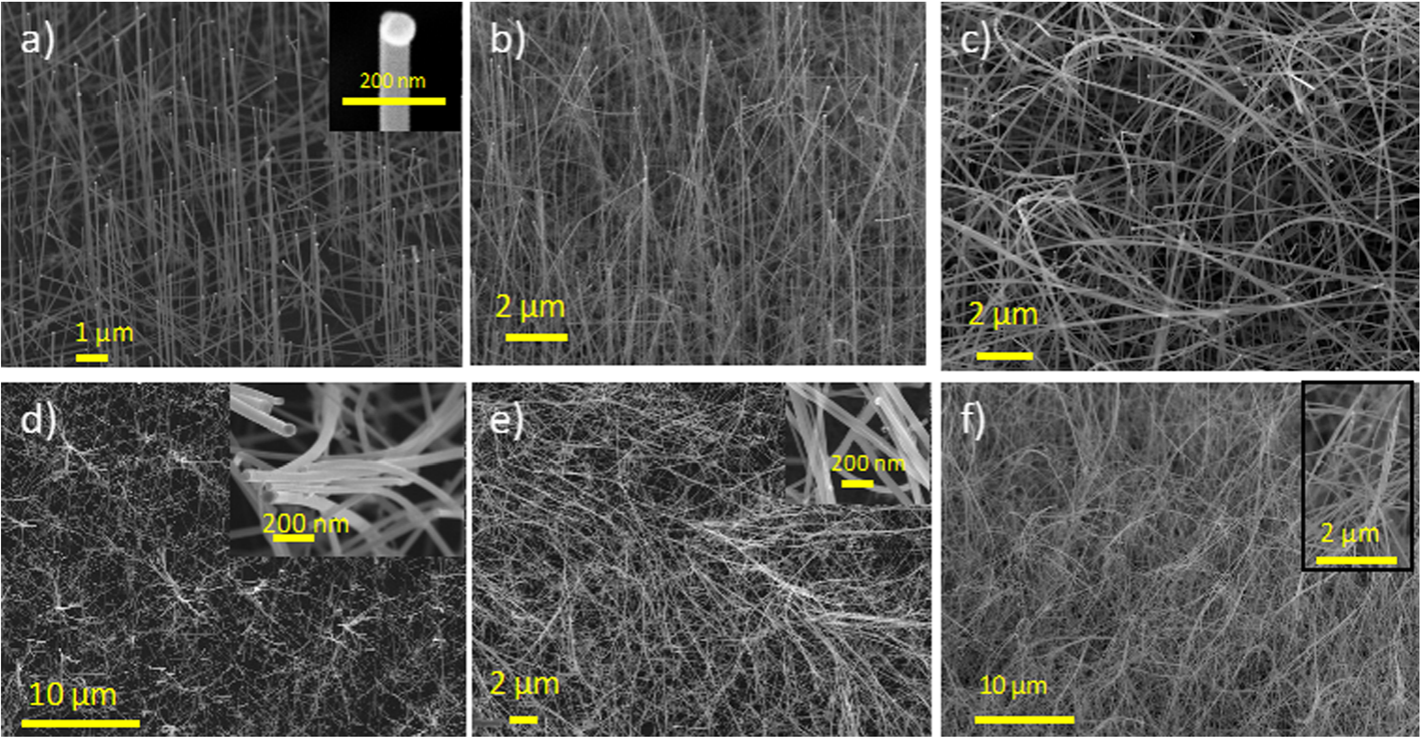
**5μm, 10μm and 20μm long SiNWs SEM images before and after charge/discharge cycling.** SEM images before charge/discharge cycling of **a**) 5 μm SiNWs, **b**) 10 μm SiNWs, **c**) 20 μm SiNWs and SEM images after charge/discharge cycling of **d**) 5 μm SiNWs, **e**) 10 μm SiNWs, **f**) 20 μm SiNWs.

**Figure 2:**
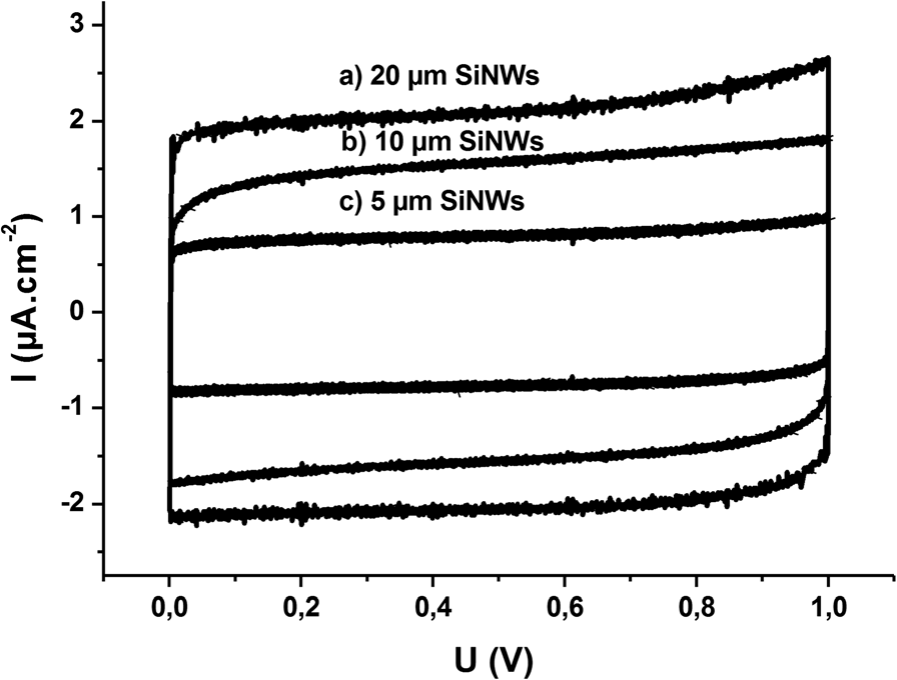
Cyclic voltammetry of symmetrical SiNWs/SiNWs micro-ultracapacitors for several SiNWs lengths.

**Figure 3:**
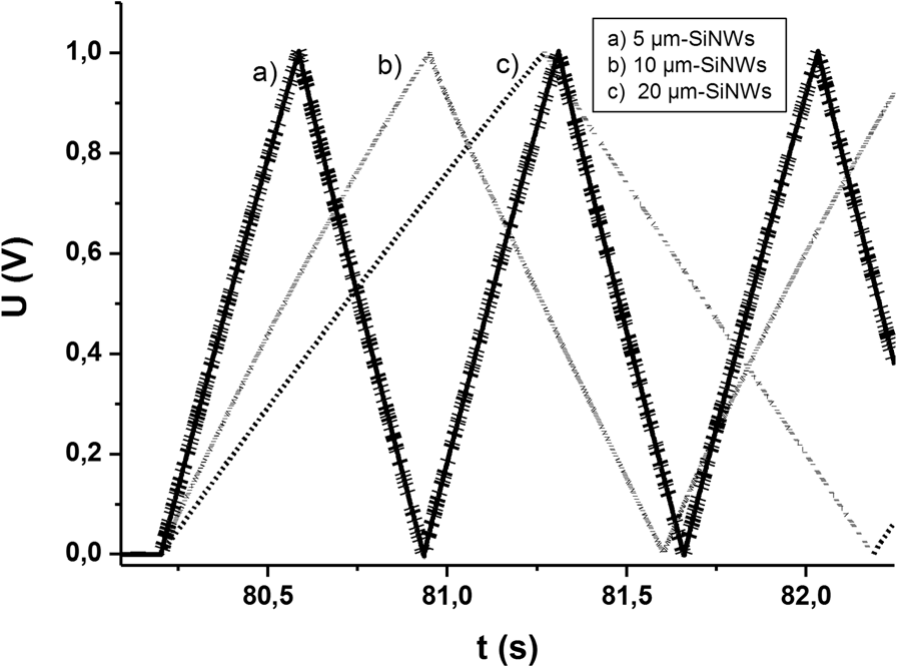
Symmetrical SiNWs/SiNWs micro-ultracapacitors Galvanostatic charge/discharge for several SiNWs lengths at ±10μA cm^−2^.

Micro-ultracapacitors with two identical n-SiNWs electrodes were built by clipping the aluminum current collector, silicon electrodes (Si = 1 cm^2^), and glass fiber paper as separator. The n-SiNWs with several lengths (5, 10, and 20 μm) were used. In the same way, a micro-EC with two bulk n-Si substrate was built. Electrochemical instruments consisted of Potentiostat/galvanostat equipped with low current channels (VMP3 from Biologic with Ec-Lab software, Slough Berkshire, UK). All SiNWs/SiNWs micro-ultracapacitors were first characterized by cyclic voltammetry with a 100 mV s^−1^ scan rate between 0.01 and 1 V (Figure 1). Then, ten galvanostatic charge/discharge cycles were performed at ±5 and ±10 μA cm^−2^ (Figure 2) and finally, all devices were cycled for 250 galvanostatic charge/discharge at 5 μA cm^−2^ (Figure 3).

## Results and discussion

### SiNWs growth by CVD

**Figure 4:**
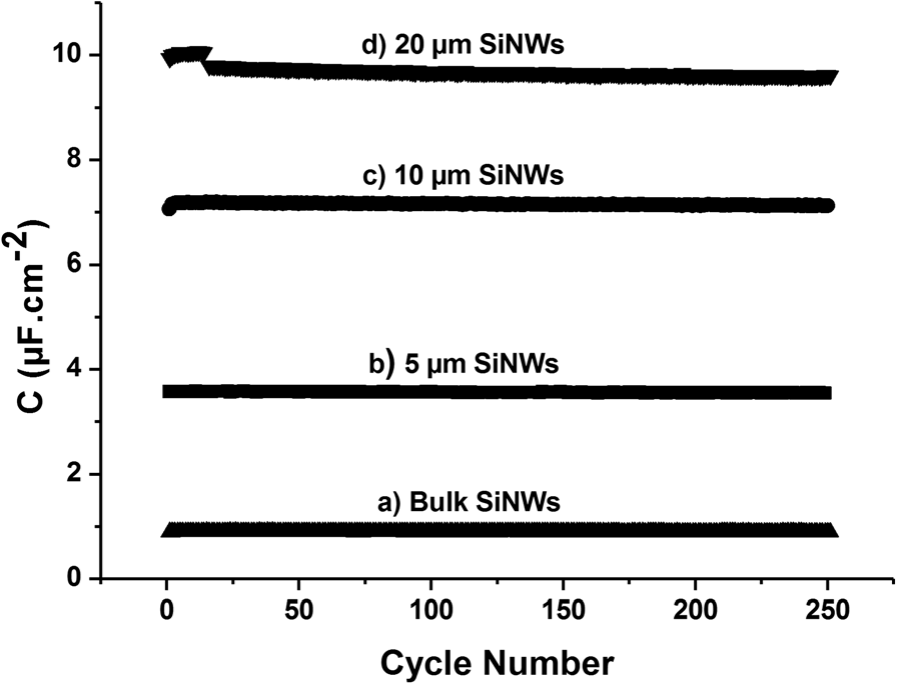
**Capacitance stability of symmetrical SiNWs/SiNWs micro-ultracapacitors during galvanostatic charge/discharge cycling at ±5 μA cm^−2^.** Capacitance stability of **a**) symmetrical bulk Si/Si micro-ultracapacitor and symmetrical SiNWs/SiNWs micro-ultracapacitors with **b**) 5 μm long SiNWs, **c**) 10 μm long SiNWs and **d**) 20 μm long SiNWs.

From 50-nm gold colloids, a NWs density of ≈3.10^8^ NWs cm^−2^, with diameters of ≈50 ± 5 nm, has been obtained for all electrodes. With growth times of 10, 20, and 40 min, electrodes with SiNWs lengths of 5 μm ± 10 nm (a in Figure 4), 10 μm ± 10 nm (b in Figure 4), and 20 μm ± 20 nm (c in Figure 4), respectively, have been obtained. Gold colloids are kept on top of the SiNWs (inserted in a in Figure 4) without any influence on the electrochemical behavior. SiNWs length, diameter, and density determination from the SEM images provides an estimation of SiNWs volume and by calculation with silicon density, an estimation of SiNWs mass (respectively, ≈12, 24, and 48 μg for 5, 10 and 20 μm NWs). The developed surface cannot be accurately determined from the SEM images. With the dopant/SiH_4_ ratio equal to 4.10^−3^ for all samples, we obtain a doping level of 4.10^19^ cm^−3^[21].

### Electrochemical characterization of SiNWs/SiNWs micro-ultracapacitors

All devices show a quasi-ideal capacitive behavior. Cyclic voltammetry curves are rectangular. Galvanostatic charge/discharge curves are triangular and symmetrical, which indicates that only very few losses occur between the charge and the discharge. An unexpected lower voltage for 1 M NEt_4_BF_4_ in PC is used to stay in the system electrochemical stability window (ESW) and avoid side reactions. In fact, the system ESW is smaller than the one obtained on platinum for this electrolyte due to silicon oxidation at a potential below the electrolyte one [15,16].

Device capacitance increase with the SiNWs length can be seen on cyclic voltammetry curves (Figure 1) and on galvanostatic charge/discharge curves (Figure 2). In fact, for the first one, capacitance is proportional to the current density difference inside the two curves (Formula 1) and for the second one it is inversely proportional to the discharge slope (Formula 2). Capacitance values have been calculated from the galvanostatic charge/discharge experiments for both current densities and reported in Table 1.

**Table 1:** SiNWs/SiNWs micro-ultracapacitors surface capacitances obtained from the galvanostatic charge/discharge (Formula 2) at 5 and 10 μA cm^−2^

**SiNWs length (μm)**	***j*** = **5μA cm**^−**2**^		***j*** = **10μA cm**^−**2**^	
	**C (μF cm**^**−2**^**)**	**C (*i *μm)/C (5 μm)**	**C (μF cm**^**−2**^**)**	**C (i μm)/C (5 μm)**
5	3.6		3.5	
10	7.2	2.0	6.7	1.9
20	9.7	2.7	9.5	2.7

Formula 1

(1) $$C=\frac{\Delta j}{2*vb}\text{,}$$

with Δ*j* as the current density differences inside the cyclic voltammetry curve and *v* as the scan rate.

Formula 2

(2) $$C=\frac{j}{\text{Discharge slope of galvanostatic charge-discharge curve}}\text{,}$$

with *j* the current density used for the galvanostatic charge/discharge.

Devices with the same SiNWs length show similar capacitance values for both current densities. As noticed on the curves, capacitance increases with SiNWs length. This increase is proportional to the length increase between 5 (≈3.5 μF cm^−2^) and 10 μm SiNWs (≈7 μF cm^−2^), but not between 5 and 20 μm (≈9.5 μF cm^−2^). This can be explained by accessible surface losses due to SiNWs constriction when substrates are stacked together. New devices avoiding this constriction will be designed and evaluated. Although previous works on the use of silicon-based electrodes for supercapacitor [10-15] reported better capacitance values, the SiNWs length influence in two electrodes devices has never been investigated. Moreover, it could be improved up to the capacitance wanted by increasing the SiNWs length and density and by improving the device design. In fact, SiNWs growth by CVD enables to tune the NWs lengths without any limitation.

Choi et al. [10] reported the use of porous SiNWs as electrode for supercapacitor in such devices but with Li^+^ containing electrolyte. Their capacitance is expressed only in force per gram, so no accurate comparison with our results is possible. Desplobain et al. [12] have obtained devices with 320 μF cm^−2^ capacitance by using gold-coated porous silicon but in aqueous electrolyte. SiNWs coated with NiO [13,14] or SiC [15] shows promising performances and cycling ability, but silicon is not the active material and their performances have not been evaluated in the two electrodes devices.

After 250 cycles at ±5 μA cm^−2^, each device shows less than 2% capacitance loss (1.8% for 20-μm SiNWs, 0.5% for 10-μm SiNWs, 0.7% for 5-μm SiNWs, and 0.5% for bulk silicon) (Figure 3). Whatever the length, SiNWs are stable after these cycling experiments, as observed on post-experimental SEM images (Figure 4). The top bending that can be observed is due to electrostatic forces occurring during the sample washing with organic solvents before the SEM observation.

Due to the moderate surface capacitance, 20-μm SiNWs-based microdevice only stores 5 μJ cm^−2^, i.e., few milliwatts per square centimeter. However, the interest of the device is more directed toward the power density which reaches 1.4 mWcm^−2^, which is close to the one of the 5-μm thick activated carbon supercapacitor (5 mW cm^−2^) [7].

## Conclusions

Highly doped SiNWs/SiNWs micro-ultracapacitors show a quasi-ideal capacitive behavior in organic electrolyte (1 M NEt_4_BF_4_ in PC). Current density used for galvanostatic charge/discharge cycling does not seem to have a major influence on the device capacitance. Devices capacitance increase with the length of the SiNWs on the electrode has been improved up to 10 μF cm^−2^ by using 20-μm SiNWs, i.e., ≈10-fold bulk silicon capacitance. This device exhibits 1.8% capacitance loss in 250 cycles with a maximum power density of 1.4 mW cm^−2^. As SiNWs growth by CVD with HCl gas enables to tune the NWs lengths without any limitation, the capacitance can be improved up to the wanted values by increasing the SiNWs length and density and by improving device design to avoid SiNWs constriction.

## Abbreviations

CVD: Chemical vapor deposition; H_2_: Dihydrogen; H_2_SO_4_: Sulfuric acid; HCl: Chloridric acid; HF: Fluoridric acid; MnO_2_: Manganese dioxide; NEt_4_BF_4_: Tetraethylammonium tetrafluoroborate; NH_4_F: Ammonium fluoride; NiO: Nickel oxide; NW: Nanowire; PC: Propylene carbonate; PH_3_: Phosphine; RuO_2_: Ruthenium dioxide; Sccm: Standard cubic centimeters; SEM: Scanning electron microscopy; Si: Silicon; SiC: Silicon carbide; SiH_4_: Silane; SiNW: Silicon nanowire; VLS: Vapor–liquid-solid.

## Competing interests

The authors declare that they have no competing interests.

## Authors’ contributions

FT carried out the SiNWs SEM characterization, the SiNWs/SiNWs ultracapacitors’ electrochemical characterization, and drafted the manuscript. NP carried out the resistivity measurements and their interpretation to determine the SiNWs doping level. TB contributed in useful discussions about results and the conception of the electrochemical study. PG developed and carried out the SiNWs growth by CVD and drafted the manuscript. SS contributed in useful discussions about results and manuscript preparation. All authors discussed the results and implications and commented on the manuscript at all stages. All the authors read and approved the final manuscript.
